# Machine learning strategy for identifying altered gut microbiomes for diagnostic screening in myasthenia gravis

**DOI:** 10.3389/fmicb.2023.1227300

**Published:** 2023-09-27

**Authors:** Che-Cheng Chang, Tzu-Chi Liu, Chi-Jie Lu, Hou-Chang Chiu, Wei-Ning Lin

**Affiliations:** ^1^PhD Program in Nutrition and Food Science, Fu Jen Catholic University, New Taipei City, Taiwan; ^2^Department of Neurology, Fu Jen Catholic University Hospital, Fu Jen Catholic University, New Taipei City, Taiwan; ^3^Graduate Institute of Biomedical and Pharmaceutical Science, Fu Jen Catholic University, New Taipei City, Taiwan; ^4^Graduate Institute of Business Administration, Fu Jen Catholic University, New Taipei City, Taiwan; ^5^Artificial Intelligence Development Center, Fu Jen Catholic University, New Taipei City, Taiwan; ^6^Department of Information Management, Fu Jen Catholic University, New Taipei City, Taiwan; ^7^School of Medicine, Fu Jen Catholic University, New Taipei City, Taiwan; ^8^Department of Neurology, Taipei Medical University, Shuang-Ho Hospital, New Taipei City, Taiwan

**Keywords:** myasthenia gravis, amplicon sequence variants, gut microbiota, machine learning, extreme gradient boosting, leave one out cross validation

## Abstract

Myasthenia gravis (MG) is a neuromuscular junction disease with a complex pathophysiology and clinical variation for which no clear biomarker has been discovered. We hypothesized that because changes in gut microbiome composition often occur in autoimmune diseases, the gut microbiome structures of patients with MG would differ from those without, and supervised machine learning (ML) analysis strategy could be trained using data from gut microbiota for diagnostic screening of MG. Genomic DNA from the stool samples of MG and those without were collected and established a sequencing library by constructing amplicon sequence variants (ASVs) and completing taxonomic classification of each representative DNA sequence. Four ML methods, namely least absolute shrinkage and selection operator, extreme gradient boosting (XGBoost), random forest, and classification and regression trees with nested leave-one-out cross-validation were trained using ASV taxon–based data and full ASV–based data to identify key ASVs in each data set. The results revealed XGBoost to have the best predicted performance. Overlapping key features extracted when XGBoost was trained using the full ASV–based and ASV taxon–based data were identified, and 31 high-importance ASVs (HIASVs) were obtained, assigned importance scores, and ranked. The most significant difference observed was in the abundance of bacteria in the *Lachnospiraceae* and *Ruminococcaceae* families. The 31 HIASVs were used to train the XGBoost algorithm to differentiate individuals with and without MG. The model had high diagnostic classification power and could accurately predict and identify patients with MG. In addition, the abundance of *Lachnospiraceae* was associated with limb weakness severity. In this study, we discovered that the composition of gut microbiomes differed between MG and non-MG subjects. In addition, the proposed XGBoost model trained using 31 HIASVs had the most favorable performance with respect to analyzing gut microbiomes. These HIASVs selected by the ML model may serve as biomarkers for clinical use and mechanistic study in the future. Our proposed ML model can identify several taxonomic markers and effectively discriminate patients with MG from those without with a high accuracy, the ML strategy can be applied as a benchmark to conduct noninvasive screening of MG.

## 1. Introduction

Myasthenia gravis (MG) is a neuromuscular junction disorder that occurs when autoantibodies bind to components of the postsynaptic muscle membrane. The most easily observed symptom is fluctuating skeletal muscle weakness (Gilhus, 2016). The development of immunomodulating treatments has significantly improved the prognosis for patients with MG (Farrugia and Goodfellow, 2020; Narayanaswami et al., 2021). Although well-established management options for MG are widely available, MG can be difficult to identify because its clinical symptoms often vary considerably and may overlap with those of other neurological disorders. Furthermore, antibody testing, which is crucial for confirming a diagnosis of MG, can be expensive, time-consuming, and not readily available and has a high rate of false negatives (Gilhus, 2016). In addition, relapse-related symptoms and their severity can vary greatly by person to person (Hehir and Silvestri, 2018). Otherwise, the severity of MG can be difficult to assess in patients with positive for acetylcholine receptor antibodies because no clear association has been established between the antibody titer and disease severity (Berrih-Aknin and Le Panse, 2014). No marker of MG has been discovered that can assist in the diagnosis, follow-up, therapy response monitoring, and clinical variability determination of the disease.

Research revealed that gut microbiomes may contain biomarkers that can be used to evaluate several neurological diseases, such as Parkinson’s disease (Lin et al., 2019). A growing body of evidence indicates that gut microbiota may be associated with immune function dysregulation, which can result in several autoimmune diseases (Pianta et al., 2017; Shahi et al., 2017; Gopalakrishnan et al., 2018; Qiu et al., 2018). Evidence also indicates that T-regulatory cells are present in large quantities in the intestinal mucosa and that microbial components and their metabolites may be involved in maintaining the homeostasis of the immune system (Chen and Tang, 2021). While several studies have demonstrated dysbiosis in autoimmune diseases, there remains a limited focus on neuromuscular disorders. Recently, there has been growing attention to the disturbance of microbiome composition and gut dysbiosis in MG, as well as its comorbidity with anxiety (Zhang et al., 2022; Kapoor et al., 2023). However, how gut microbiota alterations affect the course of such diseases remains unclear, and no method for identifying key features in gut microbiota has been discovered.

Machine learning (ML) methods, as a strategy of artificial intelligence (AI), that can successfully recognize patterns in clinical data, it can be efficiently used for triage, screening, diagnosis, and biomarker identification, and the joint use of manual and ML evaluations can offer more efficient and accurate results than the use of one method alone (Liu et al., 2019). Numerous studies have applied ML techniques to collect and analyze human microbiome data to elucidate the diverse taxonomies and functions of microbial communities and their effects on human health. However, no one-size-fits-all ML technique is available for analyzing gut microbiomes or determining which bacteria is most associated with MG. The identification of a simple screening test for the early detection of MG would allow for a timely diagnosis and the initiation of prompt treatment intervention.

Some studies have reported that the microbiota composition in the fecal samples of MG groups differed from those of healthy control groups (Moris et al., 2018; Qiu et al., 2018). Gut microbiota has been proposed as a potential diagnostic biomarker for MG therapies and early detection of progression (Kang et al., 2022; Thye et al., 2022). However, few studies have compared the feasibility and potential accuracy of applying an ML strategy to evaluate the gut microbiomes of individuals with MG. Our study hypothesized that the compositions of the gut microbiomes of individuals with and without MG would differ and that supervised ML models could be trained using gut microbiota data to provide diagnostic screening results for MG and predict clinical severity. Our study tested several ML analysis methods to identify the most favorable strategy for identifying MG. The results indicate that ML-based strategies can aid in identifying how microbiomes change in relation to MG and that the tree-based method extreme gradient boosting (XGBoost) performs the best (Chen and Guestrin, 2016). In addition, an ML-based support tool for measuring gut microbial populations was developed.

## 2. Materials and methods

### 2.1. Human subjects and sample/data collection

**Figure 1:**
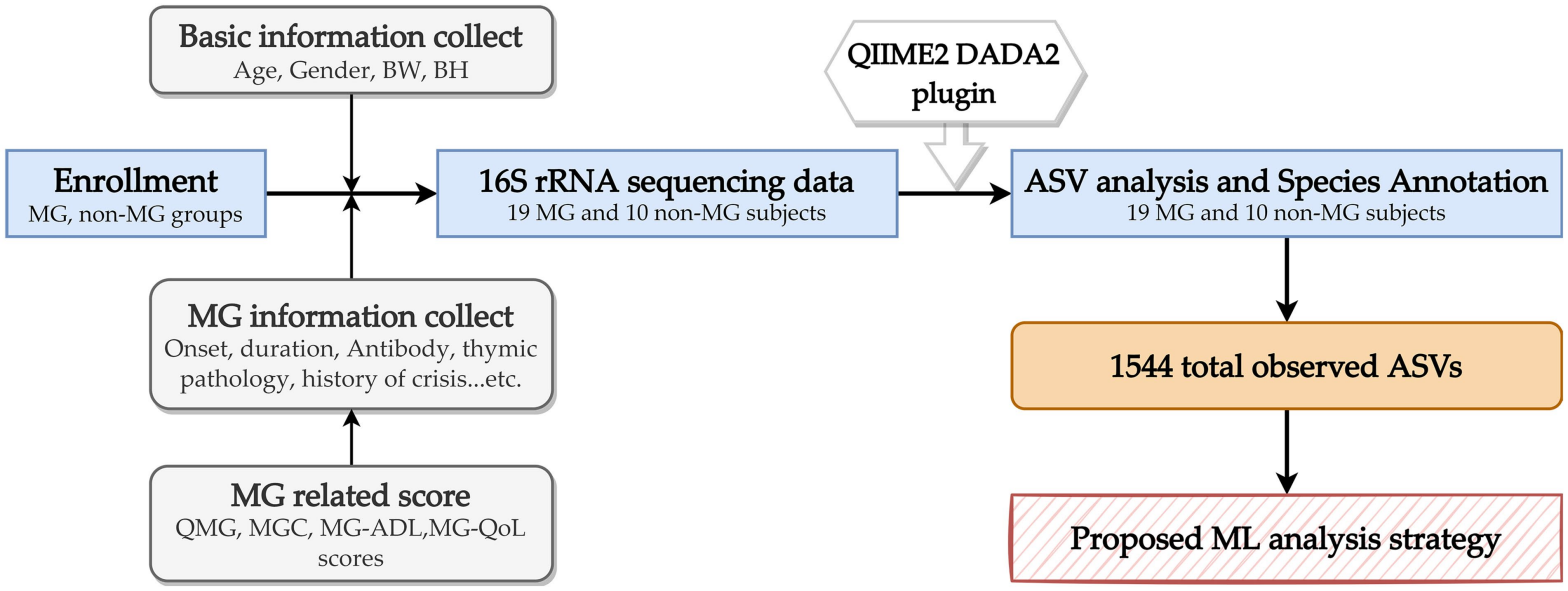
Overall study workflow. MG, myasthenia gravis; BW, body weight; BH, body height; QMG, quantitative MG score; MGC, MG composite score; MG-ADL, MG activities of daily living score; ASV, amplicon sequence variants; ML, machine learning.

In this prospective study, 19 individuals with MG and 10 individuals without were consecutively recruited from Fu-Jen Catholic University Hospital. Individuals were enrolled in the MG group if they (1) were given a diagnosis of MG on the basis of having the combination of symptoms and signs that are characteristic of muscle weakness with diurnal changes and either (2a) had a positive test result for specific autoantibodies or (2b) had a positive electrophysiological diagnosis obtained using single-fiber electromyography and repetitive nerve stimulation (Rousseff, 2021). None of the participants had received any abdominal chirurgic intervention; consumed antibiotics, probiotics, or antacids during the previous 6 months; or reported gastrointestinal symptoms during the previous year. This study was approved by the Research Ethic Committee of Fu-Jen Catholic University Hospital and written informed consent was obtained from each participant (No. FJUH109042). All experiments were completed in accordance with the Declaration of Helsinki’s Ethical Principles for Medical Research Involving Human Subjects and under a set of approved guidelines and regulations. The severity of MG was determined using quantitative MG (QMG), MG activities of daily living (MG-ADL), MG composition (MGC), and MG quality of life (MG-QoL) scores (Jaretzki et al., 2000). Using the categories of the QMG and MGC scales, we categorized the scores on these scales into ocular, bulbar, and limb groups. Figure 1 summarizes the overall study workflow.

### 2.2. Sample collection and processing

After the participants have completed the informed consent form and agreed to participate in the study, fecal samples from each volunteer were collected after enrollment. Volunteers self-collected Fresh stool samples after defecation in the hospital and immediately transferred the samples to a laboratory freezer at −80°C for cryopreservation.

Each stage in the process, including the sample testing and polymerase chain reaction (PCR) and library creation and sequencing, can affect the quality of the data, and the accuracy of analytical findings is directly influenced by the quality of data. Therefore, quality control measures were implemented at each stage of the process to ensure data accuracy.

### 2.3. DNA extraction and 16S metagenomics sequencing

Genomic DNA was extracted from the samples using the EasyPrep Stool Genomic DNA Kit (Biotools, New Taipei City, Taiwan). The DNA concentration was determined and adjusted to 5 ng/μL for subsequent processing. In accordance with the 16S Metagenomic Sequencing Library Preparation protocol (Illumina), the specific primer set 341F: 5’-CCTACGGGNGGCWGCAG-3′, 806R: 5’-GACTACHVGGGTATCTAATCC-3′ was employed to amplify the variable regions V3 and V4 of the 16S rRNA gene. A PCR was conducted using KAPA HiFi HotStart ReadyMix (Roche) and 12.5 ng of genomic DNA (gDNA) under the following conditions: 95°C for 3 min, 25 cycles of 95°C for 30 s, 55°C for 30 s, 72°C for 30 s, and a final extension of 72°C for 5 min. The reaction was subsequently maintained at 4°C. The products of the PCR were evaluated using 1.5% agarose gel, and samples with a bright main strip at approximately 500 bp were selected for further library preparation. The selected samples were purified using AMPure XP beads.

A sequencing library was prepared using the 16S Metagenomic Sequencing Library Preparation procedure (Illumina). To summarize, the 16S rRNA V3–V4 region PCR amplicon was subjected to a secondary PCR, which was conducted using the Nextera XT Index Kit with dual indices and Illumina sequencing adapters from Illumina. The indexed PCR product was evaluated for quality by using the Qubit 4.0 Fluorometer (Thermo Scientific) and a Qsep100™ system. The indexed PCR products were mixed in equal amounts to create a sequencing library. The library was sequenced on an Illumina MiSeq platform, which generated 300-bp paired reads.

### 2.4. Microbial community analysis and statistical analysis

Amplicon sequencing was performed using 300-bp paired-end raw reads, and each sample was demultiplexed on the basis of their barcode identification. Primer and adapter sequences were removed from the paired-end reads by using the QIIME2 cutadapt plugin (Martin, 2011). To construct amplicon sequence variants (ASVs), a denoising pipeline was applied using the QIIME2 DADA2 plugin (v2021.4) to implement quality filtering, dereplication, dataset-specific error model learning, denoising, paired-end-read joining, and chimera removal (Callahan et al., 2016). Trimming and filtering were performed with a maximum of two expected errors per read (maxEE = 2). The DADA2 algorithm was used to solve the problem of exact merged paired-end reads with an overlapping 12-base pair near-zero error rate. The feature-classifier and algorithm of QIIME2 was employed to annotate the taxonomic classification of each representative sequence on the basis of information retrieved from the Silva database (Bokulich et al., 2018). To analyze the sequence similarities among the ASVs, multiple sequence alignment was conducted, with the QIIME2 alignment MAFFT used against the Silva database (Katoh and Standley, 2013). A QIIME2 phylogeny fast tree was used to construct a phylogenetic tree with a set of sequences representative of the ASVs (Price et al., 2010).

### 2.5. Taxonomic analysis

The taxa that significantly differed between the MG and non-MG samples were identified, and an analysis of the overlap between the taxa of these samples was conducted. Significant biomarkers were identified through Linear discriminant analysis effect size (LEfSe) analysis (Segata et al., 2011). Subsequently, linear discriminant analysis (LDA) is applied for the bacterial taxa identified as significantly different to determine the effect size of each differentially abundant taxon. In the present study, taxa with an LDA score > 2 were considered significant.

### 2.6. Supervised ML modeling and proposed ML analytical strategy

**Figure 2:**
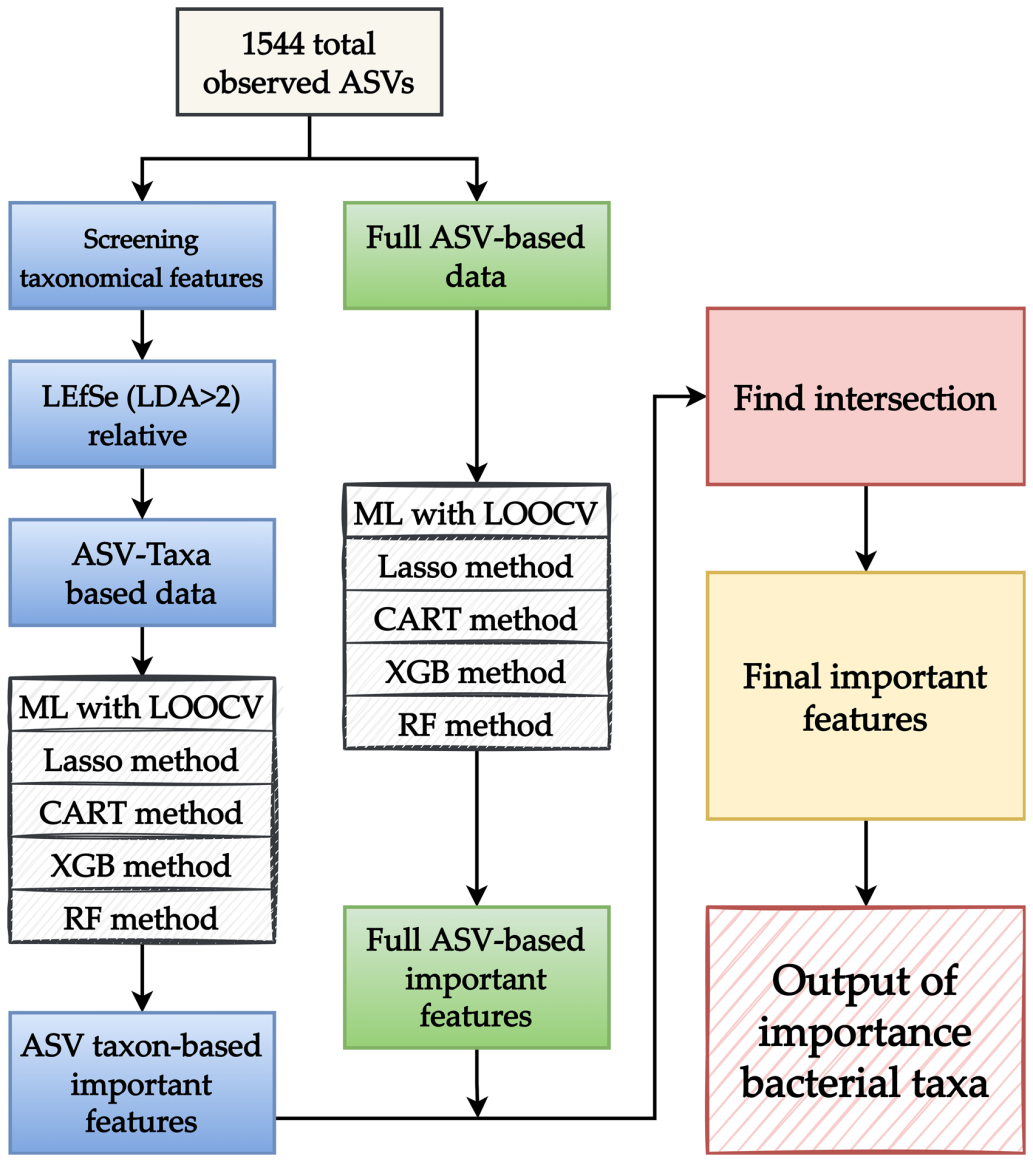
Proposed machine learning (ML) analytical strategy. After amplicon sequence variants (ASVs) are obtained, two sets of data obtained through different approaches, namely an ASV taxon–based data set (blue boxes) and full ASV–based data set (green boxes), are prepared. For the ASV taxon–based data, taxon analysis is used to screen 1,544 observed ASVs for key features. LEfSe is applied to identify taxonomic features with a linear discriminant analysis value of >2 to develop an ASV taxon–based data set. For the full ASV–based data, the 1,544 ASVs are directly used without any modifications. After the ASV taxon–based and full ASV–based data sets are created, four ML methods (Lasso, CART, XGBoost, and RF) and leave-one-out cross-validation (LOOCV) are applied, and the model with the best performance is selected. Key features are identified by applying the selected model to the key features identified in the aforementioned data sets. Because each approach provides different types of information, the overlapping key features identified in the two data sets are screened and collected to obtain a final set of key features. The output of key bacterial taxonomic features was used to identify the taxa associated with myasthenia gravis.

This study applied four ML methods, namely least absolute shrinkage and selection operator (Lasso), XGBoost, random forest (RF), and classification and regression trees (CART). Because taxonomy or ASVs-based ML approaches provide different types of information, the present study proposed an ML analytical strategy that combines the benefits and valuable information of each approach that can be used to effectively screen key taxon features. Figure 2 presents the proposed ML analytical strategy. In the strategy, two sets of data obtained using different approaches, namely ASV taxon–based data and full ASV–based data, are prepared. Four ML methods (Lasso, CART, XGBoost, and RF) and nested leave-one-out cross-validation (LOOCV) are applied to complete ML model building for each data set, and the model with the highest performance is selected. The key features of each data set are extracted, and the overlapping key features of the data sets are screened to obtain a final set of key features.

**Figure 3:**
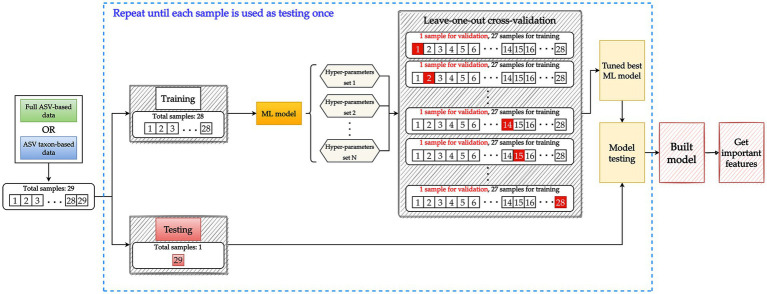
Model building involving a nested leave-one-out cross-validation (LOOCV) structure. Data are split into 28 samples for training and 1 sample for testing. To tune the hyperparameters of the ML model, the training data set with 28 samples is further split into 27 samples for training and 1 sample for validation. Each sample is used once for validation until all 28 samples have been used to validate all potential hyperparameter sets. A tuned model is then constructed using the training data (28 samples) and the best hyperparameter set. The testing data are used to evaluate the performance of the tuned model. The aforementioned process constitutes one iteration and is repeated until each sample has been used once as testing data. Key features are then extracted from the tuned model.

LOOCV was executed for the construction of each ML model. In essence, LOOCV is similar to k-fold cross-validation. The primary difference between the two is that k-fold cross-validation involves validation with one of several equally sized folds that have been randomly divided from the data whereas LOOCV involves using a single subset of the data for all rounds of the validation process (Vabalas et al., 2019). Figure 3 illustrates the nested LOOCV process used in this study.

The performance of the model was evaluated on the basis of its accuracy (ACC), precision (PRE), sensitivity (SEN), specificity (SPE) and area under the receiver operating characteristic curve (AUC). The study experiments were conducted using Python (version 3.8.8) and Jupyter Notebook (version 6.3.0) softwares (Van Rossum and Drake, 1995; Kluyver et al., 2016). XGBoost was implemented using the XGBoost package (version 1.3.3) (Chen and Guestrin, 2016), and Lasso, CART, and RF were implemented using the scikit-learn package application programming interfaces (API) (version 0.24.2) (Pedregosa et al., 2011; Buitinck et al., 2013). LOOCV and hyperparameter tuning were implemented using the scikit-learn API (Pedregosa et al., 2011).

## 3. Results

**Figure 4:**
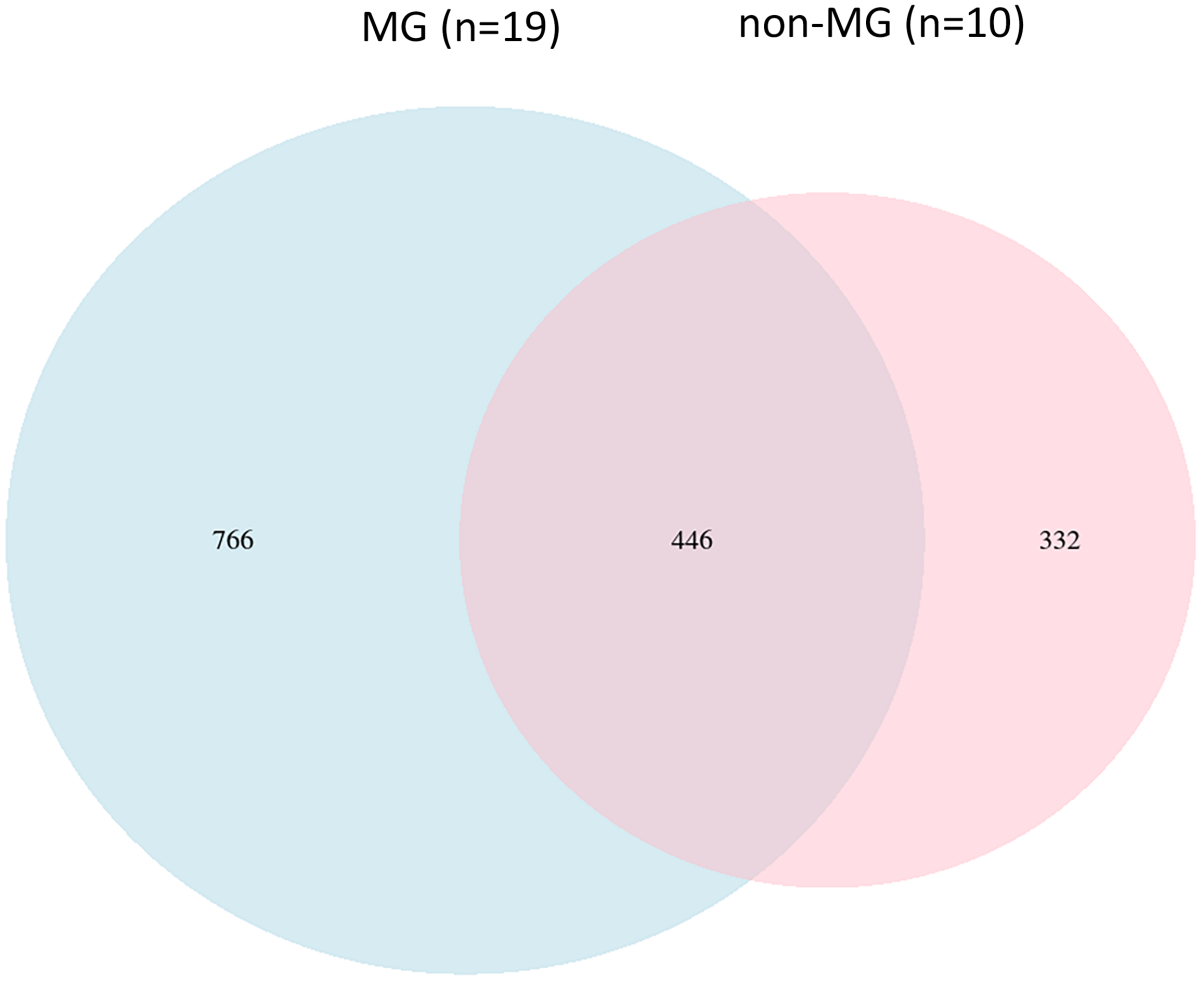
Comparison of the gut microbial composition among the two groups at ASV levels. A Venn diagram demonstrated a total of 1,544 ASVs, 446 were detected in both groups and 766, and 332 were unique to participants with (blue circle, *n* = 19) and without (pink circle, *n* = 10) MG, respectively.

Individuals who met the criteria for a diagnosis of MG were included in the present study. The mean age at enrollment was 51.5 years, and the majority of the participants were women (68%). The mean disease duration was 59.2 months. In addition, 36% of the patients with MG had a history of an MG crisis, and 21% had experienced life-threatening events at the onset of the disease. The clinical characteristics of the 19 individuals in the MG groups and 10 individuals in the non-MG group were obtained from their medical records (Table 1). The two groups did not significantly differ with respect to their age, sex, body weight, and height. To investigate the bacterial gut microbiota associated with MG, we conducted high-throughput sequencing of the V3–V4 region of the 16S ribosomal RNA gene. We obtained 1,544 ASV observations and used these ASVs to extract taxonomic information from the samples obtained from the MG and non-MG groups. A Venn diagram of the results that revealed 766 and 332 ASVs to be specific to individuals with and without MG, respectively, and 446 ASVs to be shared by individuals with and without MG (Figure 4). We also created cumulative bar charts for each taxonomic class (Supplementary Figure S1).

**Table 1:** Characteristics of subjects with MG and non-MG groups.

Characteristic	MG (*n* = 19)	Non-MG (*n* = 10)	*p*-value
Sex Female, *n* (%)	13 (68)	8 (80)	0.8212
Age (year)	51.5 ± 14.4	49.8 ± 13.9	0.7731
Height (cm)	161.4 ± 7.9	161.4 ± 4.7	0.9941
Weight (kg)	64.9 ± 15.9	63.7 ± 12.4	0.8432
BMI (kg/m^2^)	26.7 ± 4.4	24.3 ± 3.3	0.8141
Age at onset (age)	45.6 ± 14.9	–	–
Disease duration (month)	59.2 ± 77.8	–	–
Serology of AchR antibody, *n* (%)	18 (95)		
History of MG crisis, *n* (%)	7 (36)	–	–
Life threatening at onset, *n* (%)	4 (21)	–	–
Thymic pathology		–	–
Thymoma, *n* (%)	8 (42)		
Thymic hyperplasia, *n* (%)	1 (5)		
Previous Thymectomy	6 (32)	–	–
MGFA clinical class, *n* (%)		–	–
Class II	12 (63)		
Class III	4 (21)		
Class IV	3 (16)		
Daily Pyridostigmine dose (mg)	192 ± 114	–	–
PSL dose per day (mg)	9.2 ± 10.5		
IS usage, *n* (%)	2 (11)	–	–
MGQOL score	12.8 ± 13.7	–	–
QMGS	10.3 ± 4.2	–	–
QMGS – ocular	1.7 ± 1.4	–	–
QMGS – bulbar	2.4 ± 2.2	–	–
QMGS – limbs	5.1 ± 2.8	–	–
MGC	8.5 ± 8.7	–	–
MGC – ocular	1.7 ± 1.6	–	–
MGC – bulbar	5.8 ± 6.9	–	–
MGC – limbs	0.9 ± 1.6	–	–
MG-ADL	4.74 ± 4.33	–	–
Antibody titer (Nmol/L)	81.2 ± 70.8	–	–

Anti-AChR Ab, antibody against acetylcholine receptor; Anti-MuSK Ab, antibody against Muscle-specific tyrosine kinase; dSN, double seronegative; AZA, treatment with azathioprine; MMF, treatment with mycophenolate; OT, treatment with tacrolimus; IVIG, treatment with intravenous immunoglobulin; PP, treatment with plasmapheresis; PSL, prednisolone.

### 3.1. Differences in bacterial taxa between the MG and non-MG groups

**Figure 5:**
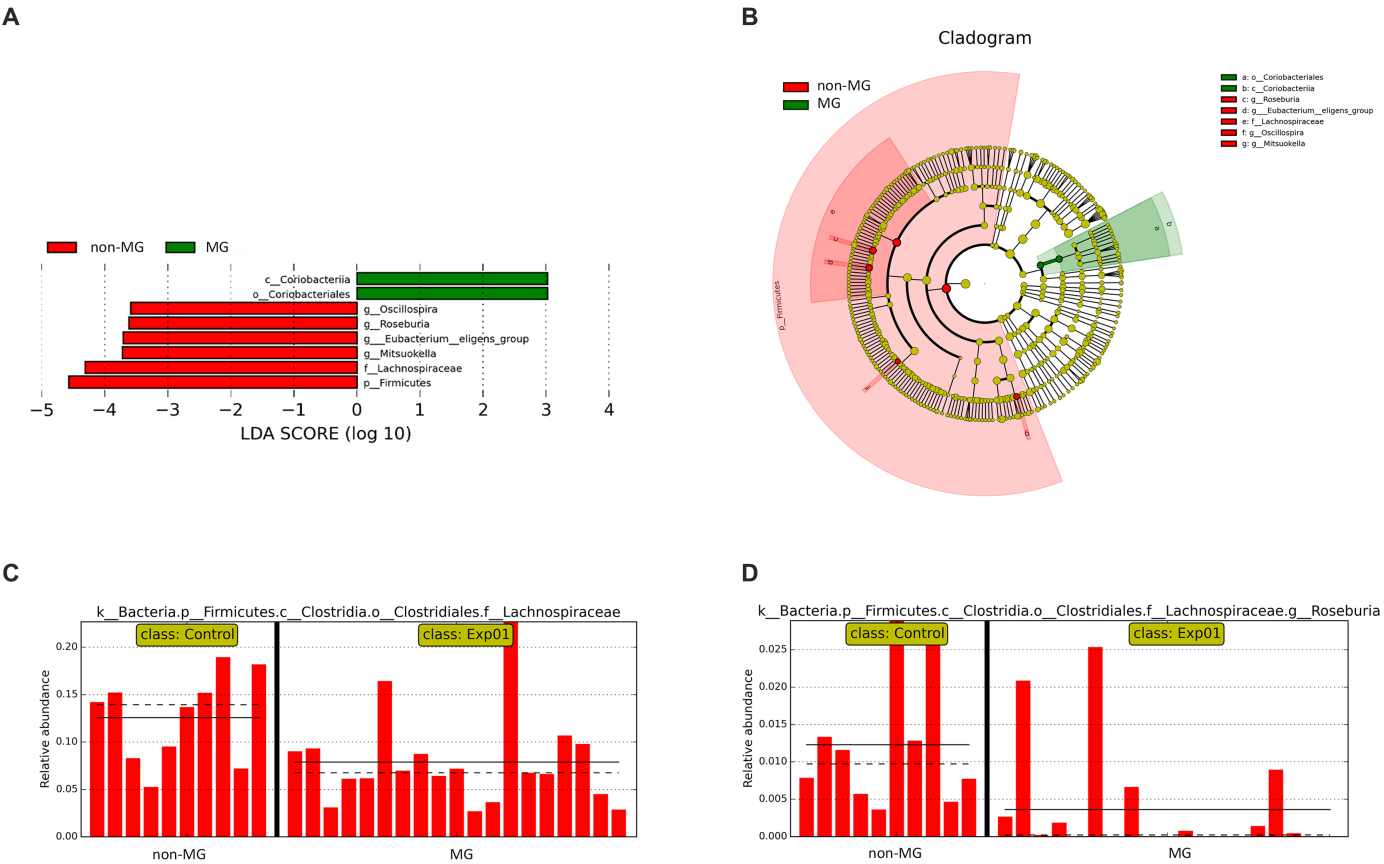
Taxonomic differences between the fecal microbiota of the MG and non-MG groups. **(A)** Cladogram created using linear discriminant analysis effect size (LEfSe) and presenting the phylogenetic distribution of the fecal microbiota of individuals with and without MG. **(B)** Linear discriminant analysis (LDA) and LEfSe revealed significant differences in the fecal microbiota of the MG (positive LDA score) and non-MG groups (negative LDA score). LDA scores (log10) > 2 are presented. **(C,D)** Representative examples of the relative abundances of *Lachnospiraceae* and *Roseburia* in individuals with and without MG, with each bar representing the abundance in a given sample. Solid and dashed lines indicate the mean and median, respectively.

To identify the significant differences in the gut microbiota between the MG and non-MG groups, we used LEfSe to identify eight taxonomic features with notable significant differences between the two groups (LDA > 2; Figures 5A,B). At the genus level, *Roseburia*, *Oscillospira*, and *Mitsuokella* were more abundant in the non-MG group (Figure 5A); at the class level, *Coriobacteriia* was more abundant in the MG group; and at the order level, *Coriobacteriales* was more abundant in the MG group. The abundances of several major bacterial taxa in the MG and non-MG groups and their phylogenetic relationships are presented in a cladogram in Figure 5B. The abundance of many species in the gut microbiomes of the MG and non-MG groups significantly differed. Figures 5C,D presents representative examples of the bacterial abundance at the family- and genus-levels in the two groups. These results support the hypothesis that the composition of gut microbiota of the MG and non-MG groups differed considerably.

### 3.2. Supervised ML analysis using enriched taxonomic features

To investigate the performance of ML methods based on different datasets, we trained supervised ML models with the taxonomic or ASV features for predictive classification and diagnostics of MG and non-MG. When enriched taxonomic features (ASV taxon–based data) were used for training, the four ML models were trained using eight taxonomic features (described above) to complete predictive classification and diagnosis of MG. Table 2 presents the performance results for the four ML models trained with ASV taxon–based data. As indicated in the table, XGBoost had the highest AUC (90.00), followed by RF (75.26), Lasso (67.89), and CART (35.26). Precision was used to measure the overall correctness of predictions of positive cases. The XGBoost model had a precision score of 100, indicating that a positive prediction by XGBoost is most likely correct. Overall, XGBoost had the highest performance when ASV taxon–based data were used for training and is thus promising as a means of correctly predicting positive cases.

**Table 2:** ML analysis using taxonomic features (ASV taxon–based ML analysis).

Method	Accuracy (%)	Precision (%)	Sensitivity (%)	Specificity (%)	AUC
Lasso	75.86	80.00	84.21	60.00	67.89
CART	41.38	66.67	21.05	80.00	35.26
XGboost	82.76	100	73.68	100	90.00
RF	75.86	100	63.16	100	75.26

AUC, area under the curve.

### 3.3. Supervised ML analysis using ASV features

The four ML models were trained with all 1,544 ASV features (full ASV–based data) to investigate the effectiveness of diagnostic classifications made on the basis of all ASVs. Table 3 presents the results. Similar to the ASV taxon–based models, the full ASV–based models were such that XGBoost had the highest AUC score (87.89), followed by RF (63.68), Lasso (56.32), and CART (46.32). In the full ASV–based model, XGBoost had a promising precision score of 100. The results indicated that XGBoost had the highest performance when the full ASV–based data were used. A comparison of the AUCs of XGBoost when ASV taxon–based data (AUC = 90.00) and full ASV–based data (AUC = 87.89) were used was conducted using the Delong test. The results revealed no statistical difference between the two (*p* = 0.43), indicating that XGBoost performed well regardless of which data set was used. Through the combination of two distinct datasets analysis, XGBoost emerges as the superior ML method for effectively distinguishing between MG and non-MG subjects. This robust outcome underscores the promising potential of ML methods in disease diagnosis within gut microbiomes.

**Table 3:** ML results when full ASV–based data (full ASV–based ML analysis).

Method	Accuracy (%)	Precision (%)	Sensitivity (%)	Specificity (%)	AUC
Lasso	72.41	76.19	84.21	50.00	56.32
CART	65.52	71.43	78.95	40.00	46.32
XGboost	86.21	100	78.95	100	87.89
RF	58.62	100	36.84	100	63.68

AUC, area under the curve.

### 3.4. XGBoost performance higher than RF on training data with enrich taxonomic and ASV features

**Figure 6:**
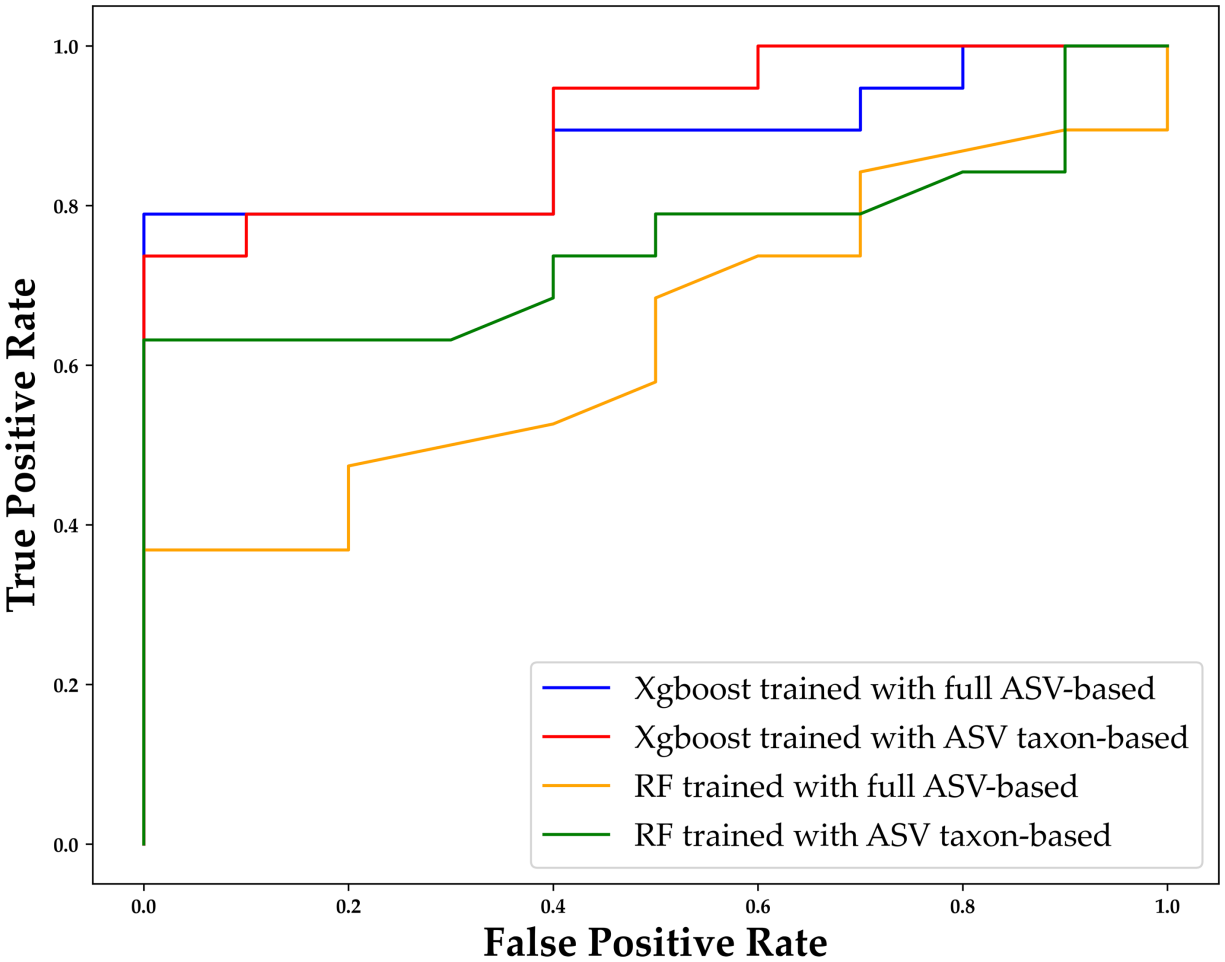
ROC curve of XGBoost and random forest (RF) with different types of data. The horizontal axis indicates the false positive rate (1–SPE), and the vertical axis indicates the true positive rate (SEN). The results for XGBoost trained with the full ASV–based and ASV taxon–based data are indicated in blue and red, respectively, and the results for RF trained with the full ASV–based and ASV taxon–based data are indicated in orange and green, respectively. ASV, amplicon sequence variant; XGBoost, extreme gradient boosting; RF, random forest.

To further assess the performance of XGBoost compared to traditional machine learning methods, we utilized the receiver operating characteristic (ROC) curve for additional verification. The performance of XGBoost remained similar when different forms of data were used as inputs (Figure 6). For purposes of comparison, RF was also included because it is commonly used in gut microbiome–related studies (Lee and Rho, 2022). The comparison of the XGBoost and RF models when different types of data were used (ASV taxon–based and full ASV–based data) revealed that XGBoost had a higher AUC than RF did, and the results were similar when the full ASV–based and ASV taxon–based data were used (Figure 6). In summary, XGBoost demonstrates high performance when trained using both general ASV data and key taxonomy features, making it a reliable tool for screening and diagnosing MG.

### 3.5. ML models trained with a combination of taxonomic and ASV features able to identify markers of MG

**Figure 7:**
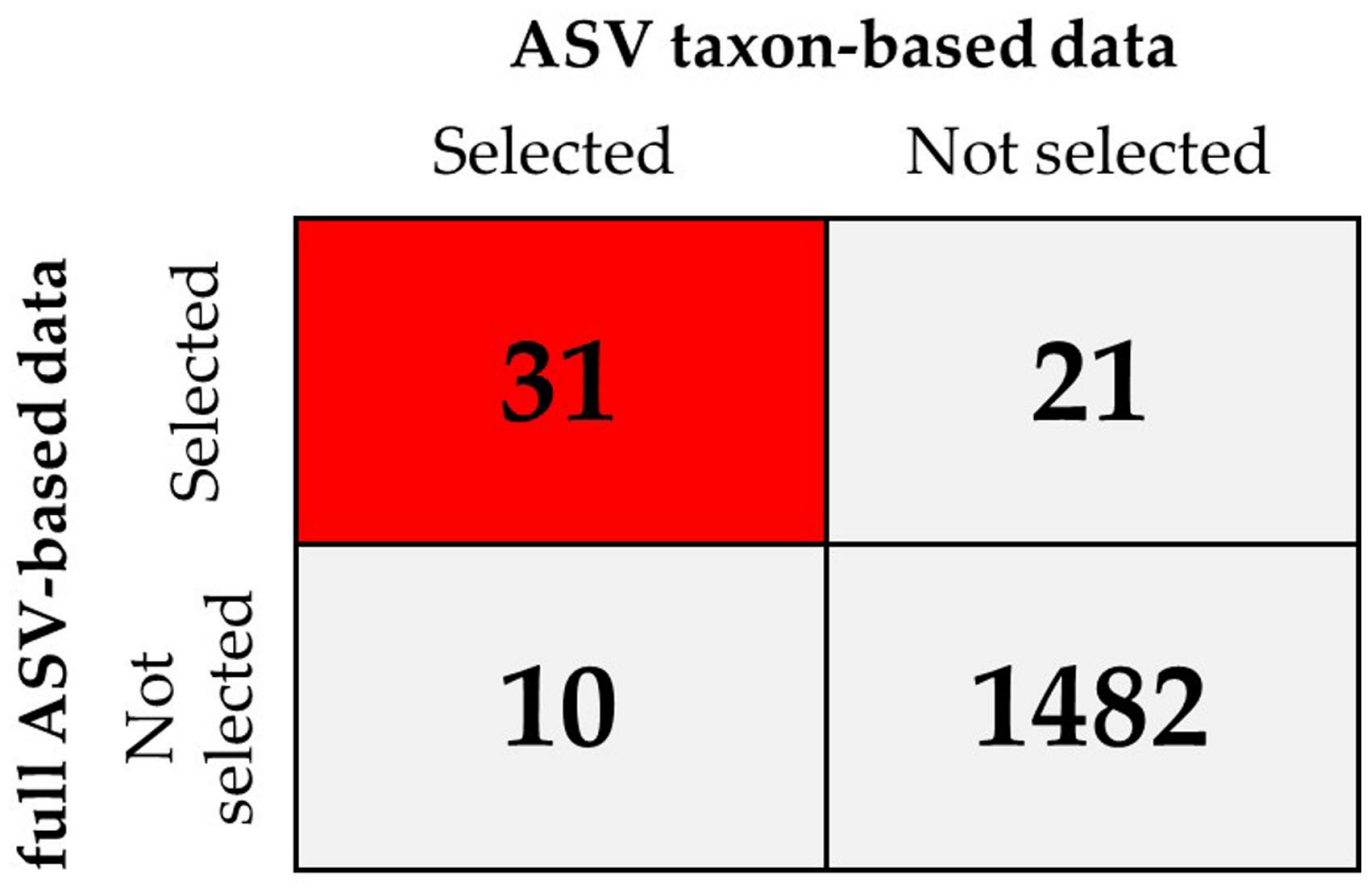
XGBoost feature selection results when the model was trained using full ASV–based and using ASV taxon–based data for comparison. The results revealed that of the 1,544 ASVs in total, 31 were selected by XGBoost when it was trained using the full ASV–based and ASV taxon–based data (red square), which indicated these were high-importance ASVs (HIASVs).

**Figure 8:**
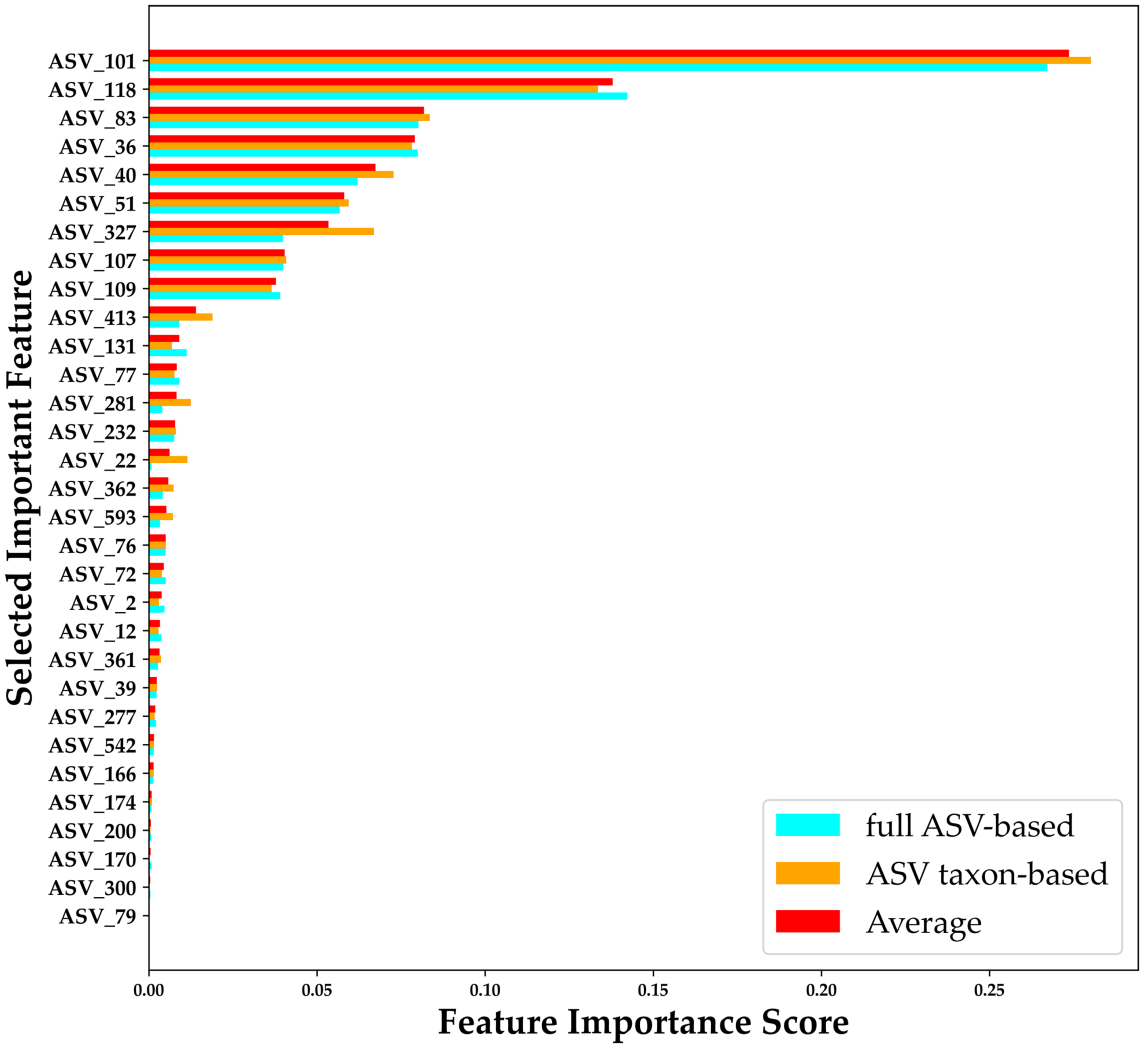
Importance scores for 31 HIASVs for classifying the presence and absence of myasthenia gravis. A comparison of the ASV feature importance score is presented in the figure, with blue indicating an importance score assigned when XGBoost trained with full ASV–based data was used, orange indicating an importance score assigned when XGBoost trained with ASV taxon–based data was used, and red indicating the average of the importance scores assigned by the Full ASV–trained and ASV taxon–trained XGBoost models. The average score was used to rank the ASVs. ASV, amplicon sequence variant.

**Figure 9:**
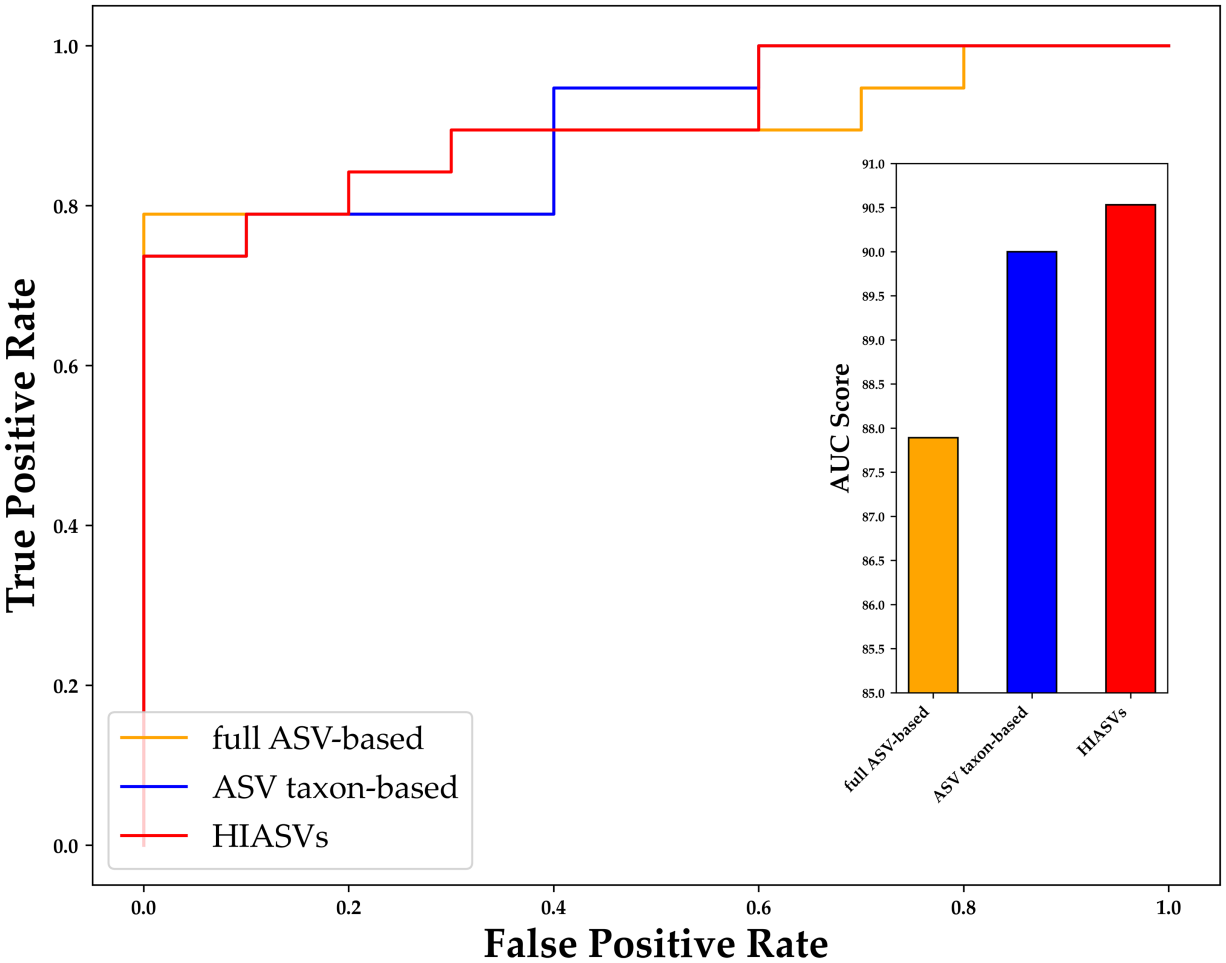
Receiver operating characteristic curve for comparing variants of XGBoost trained using different data sets. After 31 ASVs were identified as important by both XGBoost models (i.e., the model trained using the full ASV–based and that trained using ASV taxon–based data), these high-importance ASVs were used to train XGBoost, and were determined to be able to distinguish individuals with and without MG with an AUC of 90.53 (red bar), which was higher than the AUCs of the XGBoost models trained using only full ASV–based and only ASV taxon–based data. MG, myasthenia gravis; ASV, amplicon sequence variant; XGBoost, extreme gradient boosting; AUC, area under the curve.

To improve the diagnostic classification performance of the ML model, we integrated the results obtained from both the full ASV–based and ASV taxon–based datasets. The overlapping key features extracted when XGBoost was trained using the full ASV–based and ASV taxon–based data were identified and are presented in Figure 7. Thirty-one high-importance ASVs (HIASVs) were identified in the ML model when the full ASV–based and ASV taxon–based data were used. The HIASVs were assigned variable importance scores and ranked (Figure 8; Supplementary Tables S1, S2). All of the overlapping microorganisms belonged to the phylum Firmicutes. The findings revealed that the most significant difference between the gut microbiota of the individuals with and without MG was in the abundance of bacteria in the *Lachnospiraceae* and *Ruminococcaceae* families. The XGBoost algorithm was reapplied with the 31 HIASVs used to differentiate individuals with and without MG. In the XGBoost trained with the HIASVs, the dimensionality of the feature space was reduced, and the model had the highest AUC (90.53) and performed slightly better than the other ML models (Figure 9; Supplementary Table S3). The ML strategy we developed provided compelling evidence supporting our hypothesis, as it demonstrated high diagnostic classification power and generated accurate diagnostic screening results for MG.

### 3.6. Associations between gut microbiota and clinical characteristics of MG

**Figure 10:**
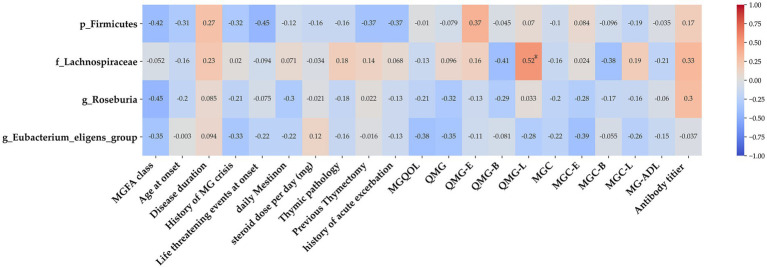
Association between gut microbiota and clinical indices of MG. Heat map of the Spearman’s rank correlation coefficient of 4 significant taxa as well as 22 clinical indices. Red squares indicate positive associations between microbial species and clinical indices; blue squares indicate negative associations. Statistical significance is indicated within the squares (*p < 0.05). The family *Lachnospiraceae* was associated with several clinical parameters. MG, myasthenia gravis; IS, immunosuppressant; MGQOL, MG quality of life; MGC, MG composite; QMGS, quantitative myasthenia gravis score; MG-ADL, MG activities of daily living.

To investigate the potential links between gut microbiome disruptions and MG clinical symptoms, a correlation analysis was conducted with a focus on the taxa of Firmicutes, *Lachnospiraceae*, *Roseburia*, and *Eubacterium*, the abundance of which was determined to significantly differ between the MG and non-MG groups. A heat map was used to present the spearman’s rank correlation coefficients of the 4 significant taxa and results on 22 clinical indices. We discovered that the abundance of *Lachnospiraceae* was generally associated with the severity of limb weakness, that is, with the limb portion of the QMG (Figure 10). These findings demonstrate that certain gut microbiota levels are associated with clinical parameters and have the potential to serve as valuable tools for assessing disease severity in the future.

## 4. Discussion

In this study, we discovered that the structures and composition of the gut microbiome were differed between MG and non-MG subjects. Among our research participants with MG, 21% had experienced a life-threatening episode upon diagnosis resulting in more severe morbidity. Additionally, 36% of the patients had a history of myasthenic crisis, indicating a potential risk of clinical deterioration in MG. Antibody titers are traditionally used to support MG evaluations but not directly correlation with clinical symptoms (Berrih-Aknin and Le Panse, 2014). Therefore, biomarkers to support MG diagnosis and disease severity screening must be identified. In the present study, the supervised ML model, XGBoost, was determined to have better performance with respect to analyzing gut microbiomes. This study’s use of LOOCV somewhat mitigated the study’s limitation of a small sample size and improved the reliability and generalizability of our findings. Our proposed ML model, which identifies several taxonomic markers, was able to effectively discriminate patients with MG from those without. Therefore, this approach has potential as a new form of ML analysis strategy for screening MG. In addition, we identified overlapping ASVs that were identified when the ML model was trained using full ASV–based and using ASV taxon–based data to select 31 HIASVs. When the model was trained using these HIASVs, the AUC was better than it was when each data set alone was used for training. Our results reveal that microbiota in the families of *Lachnospiraceae* and *Ruminococcaceae* were the most abundant in individuals with MG. We also identified microbiota potentially associated with symptoms of MG severity, that is, with limb weakness. The findings indicate that the proposed ML model based on microbiome data offers advantages and has high accuracy in identifying markers. Therefore, the model can be a potential benchmark diagnostic tool that can identify the presence of MG and gut microbiota associated with MG’s severity through noninvasive analysis.

Changes in gut microbial composition were demonstrated to affect the immunology systems that regulate bodily function. Our study revealed the differences between the microbiomes of individuals with and without MG by determining the abundance of several microbiota. The microbiota of the family *Lachnospiraceae*, a member of the phylum Firmicutes and order Clostridiales, were determined to be significantly depleted (*t* test, *p* < 0.05). Our ML models based on different ASVs verified this finding, and feature selection revealed that the family *Lachnospiraceae* was the most crucial with respect to MG. Genera from the family *Ruminococcaceae* and *Lachnospiraceae* were determined to be the most crucial for determining a diagnosis of MG when the model was trained using the HIASVs. *Lachnospiraceae* and *Ruminococcaceae* were discovered to be the two most abundant families of Clostridiales and have been reported to be associated with the maintenance of gut health and the production of short chain fatty acids (SCFAs) (Gopalakrishnan et al., 2018; Vojinovic et al., 2019). The two families are highly abundant in gut microbiota and were reported to be depleted in the gut environments of individuals with different autoimmune diseases (Biddle et al., 2013).

*Lachnospiraceae* has been indicated to potentially influence healthy gut activity, and literature reviews have revealed that different members of this family are associated with different diseases. *Lachnospiraceae* was reported to be involved in autoimmune disorders, such as multiple sclerosis and inflammatory bowel diseases (Baumgart et al., 2007; Shahi et al., 2017). However, the mechanisms underlying *Lachnospiraceae’s* regulation of immune responses and disease course remain unclear. A potential mechanism is the metabolism and production of SCFAs (Furusawa et al., 2013). This SCFA activity can modify the host immune system and function by lowering inflammatory marker levels and promoting regulatory T (Treg) cell accumulation (Atarashi et al., 2013). MG is an autoimmune condition because its pathogenesis involves disequilibrium between B cells and Treg cells, and patients with MG have a markedly lower abundance of Treg cells in their peripheral blood (Thiruppathi et al., 2012). The literature indicates that the abundance of *Ruminococcaceae* and *Lachnospiraceae* is negatively associated with these diseases (Biddle et al., 2013). A decrease in the abundance of *Lachnospiraceae* may lead to a reduction in Treg accumulation. New therapeutic strategies for treating MG should involve interventions focused on restoring *Lachnospiraceae* levels and thereby increasing Treg cell populations.

Many ML methods have been utilized in microbiota studies. ML can be used to perform numerous tasks, such as tracking phenotyping, classifying features, and identifying interactions and changes between microbiomes and other clinical variables (Gupta and Gupta, 2021; Marcos-Zambrano et al., 2021). Traditional ML models, including linear regression with Lasso and elastic nets, have been demonstrated to have higher performance in analyzing gut microbiome data and predicting dysbiosis (Pasolli et al., 2016; Lee and Rho, 2022). RF have also been used in microbiota studies. In RF models, trees are constructed to assist with decision-making and to group data into categories. In the current study, widely used ML models were used to select strategies for identifying the factors that influence MG risk (Lee and Rho, 2022). We applied XGBoost, an ensemble ML algorithm based on the decision tree method that can effectively match predicted outcomes (Chen and Guestrin, 2016). In XGBoost, many weak decision trees are integrated to form a model with strong predictive power. According to a study that compared common ML models, XGBoost, RF, and elastic nets have comparable performance when trained using microbiome data sets (Wang and Liu, 2020). In addition, XGBoost was reported to outperform a random model with respect to its cross-validation performance and to be able to forecast responses based on baseline microbiome data (Klimenko et al., 2022). Our finding that the optimal data set for training XGBoost involved both taxonomic and ASV feature data related to MG is comparable to the findings of many other studies that have investigated the characteristics that predict risk. Our results increase the depth of the understanding of the ML-XGBoost algorithm’s potential for clinically supporting disease diagnosis on the basis of gut bacterial data. The proposed XGBoost-based model may be more useful as tool for identifying the features microbiomes features and have a better accuracy and AUC than RF and Lasso models. In the future, as the number of participants increases, we can persistently substantiate this hypothesis. XGBoost could be a potential useful method in ML-based microbiomes studies.

The ML model that was trained using different taxonomic features (i.e., the ASV taxon–based data) had the same performance as that trained using the full ASV–based data. We identified the overlapping key features selected by these models to improve the ML model’s prediction power. Incorporating two sets of data to train an ML model using 31 HIASVs led to the model having the most accurate prediction. Most microbiome studies have used key operational taxonomic units to distinguish between study groups or used LDS-based taxonomic feature extraction to identify significantly different relative abundances between target groups. Our study combined genetic information (i.e., ASVs) and biological information (i.e., taxonomic features) to achieve more accurate prediction results. LOOCV was also applied and ensured that an unbiased estimate of the model’s performance was obtained because every instance in the data set is used for both training and validation. LOOCV is also more computationally expensive and particularly useful when the size of a data set is small. It allows for the data to be used to the fullest, for both training and validation (Cheng et al., 2017). Our use of LOOCV enabled us to improve the accuracy of the model’s performance and our ability to generalize our data. Furthermore, LOOCV can provide clear and interpretable results, which reduces study limitations.

Our findings are consistent with those of previous studies reporting a link between abnormalities in the gut microbiota and several autoimmune disorders (Qin et al., 2010; Chen et al., 2016; Zhou et al., 2018). Nevertheless, many autoimmune diseases do not have similar patterns of microbial dysbiosis, and therefore, the changes in the microbiota of patients with MG may not be generalizable to other autoimmune diseases. Studies have discovered that changes in gut microbiome composition can lead to inflammation that considerably affects immune responses in MG. A cohort study revealed that the gut microbiota of patients with MG was considerably altered, exhibiting a sharp decrease in the abundance of the bacterial taxa *Clostridium* correlated with a decrease in SCFA (Qiu et al., 2018). Zheng et al. demonstrated that individuals with MG often have significantly disturbed gut microbiomes and that this disturbance is associated with disease severity (Zheng et al., 2019). Another analysis revealed that MG is associated with a lower abundance of *Verrucomicrobiaceae* and *Bifidobacteriaceae* and an increased abundance of Bacteroidetes and *Desulfovibrionaceae* (Moris et al., 2018). Specially, Huang et al. found that AChR positive MG patients also experience changes in their oral microbiota (Huang et al., 2022). Our study identified bacterial genera for which the abundance differed in individuals with and without MG and applied two microbiomes-based ML models to identify key bacterial taxa. The findings may assist in improving the predictive outcomes of MG. In addition, LOOCV was used to improve the ML prediction performance. Most studies have used only OUTs or taxonomy data sets. A study reported that an ML model trained with OUTs to identify metabolite and microbiome markers was used to predict MG and that the model achieved an AUC of 0.76 (Moris et al., 2018). The model developed in our study achieved an AUC of 0.90 after being trained only with stool gut microbiome data. Stool gut microbiome data can be more easily and less expensively obtained than that of gut metabolites and metabolomes. Our findings demonstrate the potential of our proposed microbiome-based ML model as diagnostic support for identifying MG. The model can be further calibrated and the predictive capability can be improved by including more samples from different sources or stratifying particular forms of MG and data from medical records in addition to gut microbiome data. Furthermore, the significant bacterial taxonomic features identified in our study may serve as novel biomarkers for clinical use and mechanistic study in the future.

ML has shown promise in predicting outcomes and identifying biomarkers for MG. A national study used an explainable ML-based model to accurately predict short-term outcomes in MG using various clinical parameters (Zhong et al., 2023). The SHapley Additive exPlanations (SHAP) method allowed for assessing the impact of each factor on the outcome, making the results more interpretable and quantification. Supervise ML, the multinomial model has also successfully identified diagnostic biomarkers for neurological disorders, including MG, using big biological data such as genotyping, blood, and urine biochemistry data (Lam et al., 2022). During the COVID-19 pandemic, ML algorithms were utilized for telemedicine in MG, analyzing eye or body motions and vocalization for standardized data acquisition and real-time feedback (Garbey et al., 2023). In contrast to the present work, the purpose of this study was aimed to investigate fecal specimens as a simple method for MG diagnostic screening despite the absence of patient blood or genetic data and the non-use of visual computing programs, these limitations did not impact the primary objectives of the research. Although interpretability ML was not utilized to assess the impact of various microorganisms on the outcomes, the study results still hold the potential to provide valuable information for MG diagnosis. Future studies may consider increasing the number of participants, incorporating blood and genetic data, and exploring the use of interpretable machine learning models to gain deeper insights into the influence of microbiota on MG.

Our study has some limitations. First, the numbers of recruited subjects were small and only from a single geographic region with lack of ancestry data, which limiting our ability to analyze potential confounding factors. Although we applied LOOCV to improve our model’s prediction, additional large, multi-national and multi-center cohort studies should be conducted to validate our results. Second, the medication status of the recruited patients with MG differed, which could have affected the microbial compositions of their guts. Third, we did not analyze the metabolome of the stool sample. Gut microbiotas changes cannot provide the total necessary quantitative functional state of the microbiomes (Zierer et al., 2018). Forth, we did not record the dietary status of the participants. Based on the literature review, dietary is indeed a crucial factor influencing gut microbiota composition (Leeming et al., 2019; Zhang, 2022). Therefore, future research should incorporate participants’ dietary records as a basis. Fifth, the proportion of males (32%) was relatively fewer in number. MG has been known to affect females more prominently (Jayam Trouth et al., 2012). The peaks was around at age 30 and 50 (Carr et al., 2010). Therefore, most of the research on MG and gut microbiota is based on female populations (Zheng et al., 2019; Tan et al., 2020). However, the limited number of male samples can be considered a limitation in the search for biomarkers. Finally, our study did not determine whether dysbiosis is the consequence, cause, or both of MG. Future longitudinal, multi-center, large cohort studies should be conducted, combing the recording of dietary and the ancestry data with a focus on the pathophysiology of bacterial taxa involved in MG. Additional research should be performed to identify the specific microbial species associated with MG and their corresponding metabolites to assist in defining targets for MG therapy.

## 5. Conclusion

Our study is the first to demonstrate the potential for using artificial intelligence through ML modeling to complete convenient diagnostic screening of MG on the basis of fecal microbiota composition. Our gut microbiome-based ML strategy can be used as a screening method to support the diagnosis and progression of MG. In addition, the combination ML-based feature selection approaches expand the knowledge on the biomarkers of MG. XGboost-based feature selection identified of HIASVs not only reduced the computational complexity of the ML model but also improved its diagnostic classification performance. These HIASVs may serve as novel biomarkers for clinical and mechanistic study in the future. Taken together, our findings provided a novel and user-friendly ML-based algorithm for explore critical microbiomes and diagnostic tools in MG. Future studies should prioritize conducting longitudinal, multi-center research to deepen the understanding of the mechanisms involved in the interactions of ASVs with hosts, which will aid in defining targets for MG therapy.

## Data availability statement

The datasets presented in this study can be found in online repositories. The names of the repository/repositories and accession number(s) can be found below: NCBI – https://datadryad.org/stash/share/GewdUVu1bh5x0KNldA2E9qlN9ryGurFOCOdV-pKpLzk.

## Ethics statement

The studies involving humans were approved by Research Ethic Committee of Fu-Jen Catholic University Hospital. The studies were conducted in accordance with the local legislation and institutional requirements. The participants provided their written informed consent to participate in this study.

## Author contributions

C-CC and W-NL was involved in the study design, conducted the experiments, and writing the first draft of the paper. C-CC and H-CC were responsible for data collection. W-NL was responsible for proofreading and paper revision. C-CC, T-CL, and C-JL conducted the experiments, analyzed and interpreted the data. All authors have read and agreed to the published version of the manuscript.
