# Supplementary material for: Machine learning strategy for identifying altered gut microbiomes for diagnostic screening in myasthenia gravis
Source: Front Microbiol. 2023 Sep 27;14:1227300. doi: 10.3389/fmicb.2023.1227300 (PMC10565662; doi:10.3389/fmicb.2023.1227300)
Supplement: Supplementary file 1 [file Data_Sheet_1.docx]

Supplementary Material

Machine learning strategy for identifying altered gut microbiomes for diagnostic screening in myasthenia gravis

Che-Cheng Chang, Tzu-Chi Liu, Chi-Jie Lu, Hou-Chang Chiu and Wei-Ning Lin

*** Correspondence:** Wei-Ning Lin: 081551@mail.fju.edu.tw

# Supplementary Figures and Tables

## Supplementary Figures


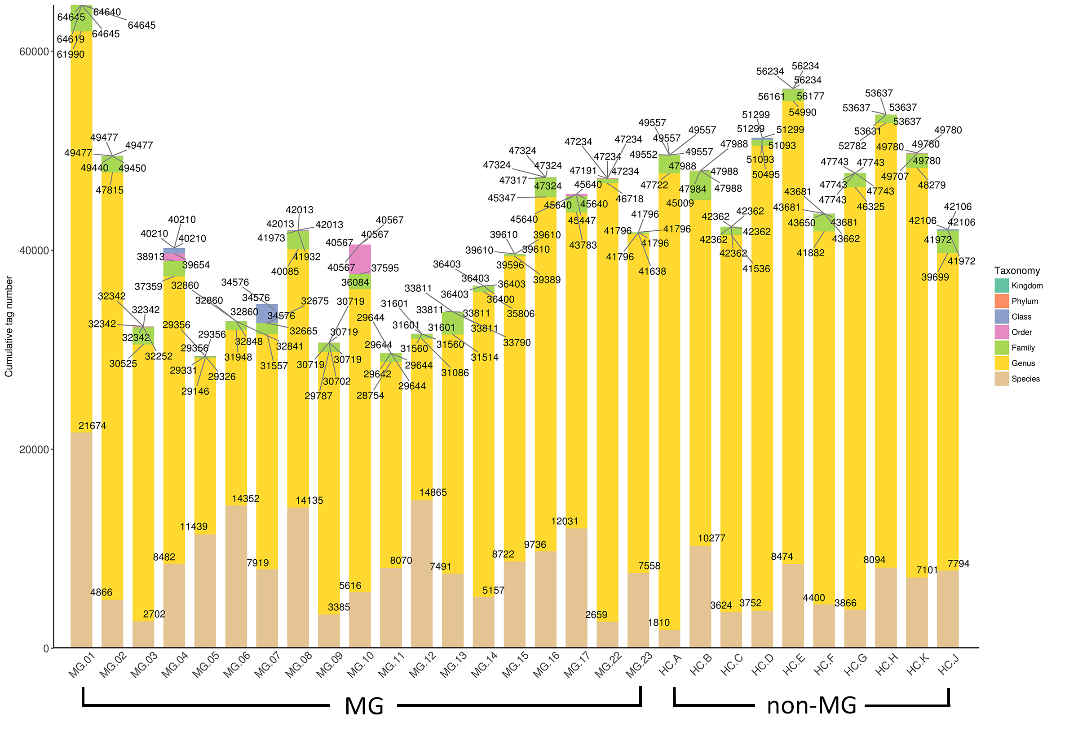


**Supplementary Figure 1.** Cumulative bar chart for all taxonomic classes depicting the abundance of the bacterial phyla, families, and genera in the guts of individuals with and without myasthenia gravis.

## Supplementary Tables

**Supplementary Table 1.** Score ranking results for 31 features selected through XGBoost trained using both full ASV–based and ASV taxon–based data.

| Ranking | ASV features | Full ASVs ML  importance score | ASV-Taxa ML  importance score | Average  importance score |
| --- | --- | --- | --- | --- |
| 1 | ASV_101 | 26.72 | 28.02 | 27.37 |
| 2 | ASV_118 | 14.23 | 13.36 | 13.79 |
| 3 | ASV_83 | 8.02 | 8.35 | 8.18 |
| 4 | ASV_36 | 8.00 | 7.82 | 7.91 |
| 5 | ASV_40 | 6.21 | 7.28 | 6.74 |
| 6 | ASV_51 | 5.68 | 5.94 | 5.81 |
| 7 | ASV_327 | 3.99 | 6.70 | 5.34 |
| 8 | ASV_107 | 4.00 | 4.08 | 4.04 |
| 9 | ASV_109 | 3.91 | 3.65 | 3.78 |
| 10 | ASV_413 | 0.90 | 1.89 | 1.40 |
| 11 | ASV_131 | 1.13 | 0.69 | 0.91 |
| 12 | ASV_77 | 0.91 | 0.76 | 0.83 |
| 13 | ASV_281 | 0.40 | 1.25 | 0.82 |
| 14 | ASV_232 | 0.76 | 0.81 | 0.78 |
| 15 | ASV_22 | 0.08 | 1.15 | 0.61 |
| 16 | ASV_362 | 0.41 | 0.74 | 0.57 |
| 17 | ASV_593 | 0.33 | 0.72 | 0.52 |
| 18 | ASV_76 | 0.50 | 0.51 | 0.50 |
| 19 | ASV_72 | 0.50 | 0.39 | 0.44 |
| 20 | ASV_2 | 0.47 | 0.30 | 0.38 |
| 21 | ASV_12 | 0.37 | 0.29 | 0.33 |
| 22 | ASV_361 | 0.28 | 0.36 | 0.32 |
| 23 | ASV_39 | 0.23 | 0.24 | 0.24 |
| 24 | ASV_277 | 0.21 | 0.17 | 0.19 |
| 25 | ASV_542 | 0.15 | 0.16 | 0.15 |
| 26 | ASV_166 | 0.14 | 0.14 | 0.14 |
| 27 | ASV_174 | 0.08 | 0.09 | 0.08 |
| 28 | ASV_200 | 0.07 | 0.05 | 0.06 |
| 29 | ASV_170 | 0.08 | 0.02 | 0.05 |
| 30 | ASV_300 | 0.05 | 0.03 | 0.04 |
| 31 | ASV_79 | 0.01 | 0.01 | 0.01 |

Abbreviation: ASV, amplicon sequence variant; ML, machine learning

**Supplementary Table 2.** Ranking of importance of bacterial taxa that overlapped when XGBoost was trained with full ASV–based and ASV taxon–based data.

| Ranking | ASV number | Phylum | Order | Family | Genus |
| --- | --- | --- | --- | --- | --- |
| 1 | ASV_101 | *Firmicutes* | *Clostridiales* | *Lachnospiraceae* | *Eubacterium eligens group* |
| 2 | ASV_118 | *Firmicutes* | *Clostridiales* | *Lachnospiraceae* |  |
| 3 | ASV_83 | *Firmicutes* | *Clostridiales* | *Lachnospiraceae* | *Roseburia* |
| 4 | ASV_36 | *Firmicutes* | *Selenomonadales* | *Veillonellaceae* | *Megasphaera* |
| 5 | ASV_40 | *Firmicutes* | *Clostridiales* | *Lachnospiraceae* | *Agathobacter* |
| 6 | ASV_51 | *Firmicutes* | *Clostridiales* | *Lachnospiraceae* |  |
| 7 | ASV_327 | *Firmicutes* | *Clostridiales* | *Lachnospiraceae* | *Lachnospiraceae UCG 010* |
| 8 | ASV_107 | *Firmicutes* | *Clostridiales* | *Lachnospiraceae* | *Eubacterium eligens group* |
| 9 | ASV_109 | *Firmicutes* | *Clostridiales* | *Lachnospiraceae* | *Lachnoclostridium* |
| 10 | ASV_413 | *Firmicutes* | *Clostridiales* | *Ruminococcaceae* | *Oscillospira* |
| 11 | ASV_131 | *Firmicutes* | *Clostridiales* | *Lachnospiraceae* | *Lachnospiraceae UCG 004* |
| 12 | ASV_77 | *Firmicutes* | *Clostridiales* | *Lachnospiraceae* | *Roseburia* |
| 13 | ASV_281 | *Firmicutes* | *Clostridiales* | *Lachnospiraceae* | *Eubacterium eligens group* |
| 14 | ASV_232 | *Firmicutes* | *Clostridiales* | *Ruminococcaceae* | *UBA1819* |
| 15 | ASV_22 | *Firmicutes* | *Clostridiales* | *Ruminococcaceae* | *Faecalibacterium* |
| 16 | ASV_362 | *Firmicutes* | *Clostridiales* | *Ruminococcaceae* | *Phocea* |
| 17 | ASV_593 | *Firmicutes* | *Clostridiales* | *Ruminococcaceae* | *Flavonifractor* |
| 18 | ASV_76 | *Firmicutes* | *Clostridiales* | *Lachnospiraceae* | *Lachnoclostridium* |
| 19 | ASV_72 | *Firmicutes* | *Clostridiales* | *Ruminococcaceae* | *Oscillibacter* |
| 20 | ASV_2 | *Firmicutes* | *Selenomonadales* | *Acidaminococcaceae* | *Phascolarctobacterium* |
| 21 | ASV_12 | *Firmicutes* | *Clostridiales* | *Ruminococcaceae* | *Faecalibacterium* |
| 22 | ASV_361 | *Firmicutes* | *Clostridiales* | *Ruminococcaceae* | *Oscillibacter* |
| 23 | ASV_39 | *Firmicutes* | *Clostridiales* | *Lachnospiraceae* | *Lachnospira* |
| 24 | ASV_277 | *Firmicutes* | *Clostridiales* | *Lachnospiraceae* | *Dorea* |
| 25 | ASV_542 | *Firmicutes* | *Clostridiales* | *Ruminococcaceae* | *Intestinimonas* |
| 26 | ASV_166 | *Firmicutes* | *Clostridiales* | *Ruminococcaceae* | *Ruminococcaceae UCG 013* |
| 27 | ASV_174 | *Firmicutes* | *Clostridiales* | *Lachnospiraceae* |  |
| 28 | ASV_362 | *Firmicutes* | *Clostridiales* | *Ruminococcaceae* | *Phocea* |
| 29 | ASV_413 | *Firmicutes* | *Clostridiales* | *Ruminococcaceae* | *Oscillospira* |
| 30 | ASV_542 | *Firmicutes* | *Clostridiales* | *Ruminococcaceae* | *Intestinimonas* |
| 31 | ASV_593 | *Firmicutes* | *Clostridiales* | *Ruminococcaceae* | *Flavonifractor* |

Abbreviation: ASV, amplicon sequence variant; ML, machine learning

**Supplementary Table 3.** Results of XGBoost with different forms of data.

| **Data Based** | **Number of ASVs** | **ACC** | **SEN** | **SPE** | **AUC** |
| --- | --- | --- | --- | --- | --- |
| Full ASV | 891 | 86.21 | 78.95 | 100 | 87.89 |
| ASV Taxon | 1544 | 82.76 | 73.68 | 100 | 90.00 |
| HIASVs | 31 | 82.76 | 73.68 | 100 | 90.53 |

Abbreviation: ASV, amplicon sequence variant; HIASV, high-importance ASV; ACC, accuracy; SEN, sensitive; SPE, specificity; AUC, area under the curve.
